# Supplementary material for: The Ectomycorrhizal Fungi and Soil Bacterial Communities of the Five Typical Tree Species in the Junzifeng National Nature Reserve, Southeast China
Source: Plants (Basel). 2023 Nov 14;12(22):3853. doi: 10.3390/plants12223853 (PMC10675191; doi:10.3390/plants12223853)
Supplement: Supplementary file 1 [file plants-12-03853-s001.zip › plants-2678611-supplementary.pdf]

Supplementary Files

1. Supplementary Tables

Supplementary Table S1. General information on the five typical forests in the reserve.

Tree stand type	Latitude	Longitude	Altitude (m)	Tree composition	Forest type
PM	117°28′28″N	26°21′39″E	712	*Pinus massoniana*	Natural forest
CC	117°28′26″N	26°21′39″E	766	*Castanopsis carlesii*	Natural forest
CE	117°29′32″N	26°20′44″E	766	*Castanopsis eyrei*	Natural forest
CF	117°29′24″N	26°20′59″E	753	*Castanopsis fargesii*	Natural forest
KC	117°28′27″N	26°21′48″E	783	*Keteleeria cyclolepis*	Natural forest

Pinus massoniana (PM), *Castanopsis carlesii* (CC), *Castanopsis eyrei* (CE), *Castanopsis fargesii* (CF), *Keteleeria cyclolepis* (KC).

Supplementary Table S2. Soil properties of five tree species.

Forest type	pH	SOM ($g \cdot kg^{-1}$)	TP ($g \cdot kg^{-1}$)	TK ($g \cdot kg^{-1}$)	TOC ($g \cdot kg^{-1}$)	DTN ($g \cdot kg^{-1}$)
PM	3.61±0.13c	58.74±1.57a	0.28±0.02b	9.6±0 .75c	1±0.02a	0.09±0.01c
CC	4.23±0.13a	51.74±0.85b	0.11±0.02c	14.39±1.38b	0.73±0.09bc	0.11±0.02c
CE	3.45±0.12c	51.59±1.1b	0.43±0.03a	8.19±0.46c	0.66±0.13bc	0.21±0.03a
CF	4.29±0.03a	50.98±2.1b	0.16±0.03c	30.59±1.17a	0.57±0.05c	0.1±0.01c
KC	3.84±0.05b	56.91±1.18a	0.45±0.02a	8.17±0.42c	0.87±0.18ab	0.16±0.03b

Soil potential hydrogen (pH), soil organic matter (SOM), total phosphorus (TP), total potassium (TK), dissolved organic carbon (DOC), dissolved organic nitrogen (DON); *Pinus massoniana* (PM), *Castanopsis carlesii* (CC), *Castanopsis eyrei* (CE), *Castanopsis fargesii* (CF), *Keteleeria cyclolepis* (KC); different letters indicate significant differences ($P < 0.05$) among five forests based on a one-way ANOVA followed by Tukey test.

Supplementary Table S3. Identities of ectomycorrhizal fungi based on the DNA sequence BLAST results of the five tree species.

No.	Taxonomy	GenBank Accession Number	Sequence length (bp)	Closest GenBank match	% Match
1	*Amanita pseudosychnopyramis*	OR258991	637	Amanita pseudosychnopyramis OM510279	100
2	*Cenococcum geophilum*	OR258990	447	Cenococcum geophilum LC095091	100
3	*Cortinarius carneoroseus*	OR259001	490	Cortinarius carneoroseus OP339601	97.2
4	Cortinariaceae sp. I	OR258999	579	Cortinarius triangulus NR171373	89.18
5	Cortinariaceae sp. II	OR259000	558	Cortinarius ochrophyllus OM001518	86.63
6	*Hortiboletus rubellus*	OR259007	583	Boletus rubellus voucher GQ166883	98.46
7	Helotiales sp.	OR259005	487	Helotiales sp. LC218312	100

8	*Inocybe posterula*	OR258998	780	Inocybe posterula HQ604298	99.74
9	*Lactarius atrofuscus*	OR259002	522	Lactarius atrofuscus MK351919	97.75
10	*Lactarius vividus*	OR259003	578	Lactarius vividus voucher MT568545	97.46
11	*Russula aff. kansaiensis*	OR258996	630	Russula aff. kansaiensis 2C7 AB973773	97.75
12	*Russula compacta*	OR258994	501	Russula compacta MK430040	99.40
13	*Russula xerampelina*	OR258995	540	Russula xerampelina MW517291	100
14	*Russula* sp.	OR258997	537	Russula sp. EF627043	98.70
15	*Sebacina* sp.	OR259006	605	uncultured Sebacina MF405785	99.83
16	*Tomentella* sp.	OR258993	610	uncultured Tomentella GQ240905	98.69
17	Trichocomaceae sp.	OR258992	517	Trichocomaceae sp. JX243820	93.67
18	Thelephoraceae sp.	OR259004	704	Thelephoraceae sp. MT522580	98.90

Supplementary Table S4. Sorensen (bottom left) and Jaccard similarity index (top right) of EMF in five tree species**.**

**Tree species**	**Similarity index**				
	**PM**	**CC**	**CE**	**CF**	**KC**
PM		0.12	0.17	0.17	0.10
CC	0.27		0.07	**0.19**	0.08
CE	0.40	0.15		0.11	0
CF	0.40	**0.46**	0.25		0.13
KC	0.22	0.17	0	0.29	

Supplementary Table S5. Relative frequency (RF), relative abundance (RA), and importance value (IV) of ECM fungi in the five tree species at the species levels.

**Identification**	**Relative frequency（RF)**					**Relative abundance (RA)**					**Importance value (IV)**				
	**PM**	**CC**	**CE**	**CF**	**KC**	**PM**	**CC**	**CE**	**CF**	**KC**	**PM**	**CC**	**CE**	**CF**	**KC**
Amanita pseudosychnopyramis	-	7.14	-	-	-	-	1.23	-	-	-	-	4.19	-	-	-
Cenococcum geophilum	36.36	21.43	-	33.33	40	13.03	26.72	-	34.24	66.97	24.7	24.08	-	33.79	53.48
Cortinarius carneoroseus	-	-	22.22	-	-	-	-	1.41	-	-	-	-	11.82	-	-
Cortinariaceae sp. I	-	7.14	-	33.33	-	-	4.93	-	3.2	-	-	6.03	-	18.27	-
Cortinariaceae sp. II	9.09	21.43	44.44	8.33	-	22.41	47.17	87.44	2.33	-	15.75	34.3	65.94	5.33	-
Hortiboletus rubellus	-	14.29	-	-	-	-	6.16	-	-	-	-	10.22	-	-	-
Helotiales sp.	-	7.14	-	-	-	-	1.23	-	-	-	-	4.19	-	-	-
Inocybe posterula	9.09	-	-	-	-	29.06	-	-	-	-	19.08	-	-	-	-
Lactarius atrofuscus	9.09	-	11.11	-	-	8.02	-	9.73	-	-	8.55	-	10.42	-	-
Lactarius vividus	27.27	-	-	-	-	12.53	-	-	-	-	19.9	-	-	-	-

Russula aff. kansaiensis	-	7.14	-	-	-	-	3.69	-	-	-	-	5.42	-	-	-
Russula compacta	-	-	-	-	20	-	-	-	-	4.58	-	-	-	-	12.29
Russula xerampelina	-	-	-	25	-	-	-	-	60.23	-	-	-	-	42.61	-
Russula sp.	-	7.14	-	-	-	-	2.46	-	-	-	-	4.8	-	-	-
Sebacina sp.	-	-	22.22	-	-	-	-	1.41	-	-	-	-	11.82	-	-
Tomentella sp.	-	-	-	-	40	-	-	-	-	28.45	-	-	-	-	34.22
Trichocomaceae sp.	-	7.14	-	-	-	-	6.4	-	-	-	-	6.77	-	-	-
Thelephoraceae sp.	9.09	-	-	-	-	14.96	-	-	-	-	12.03	-	-	-	-

Pinus massoniana (PM), *Castanopsis carlesii* (CC), *Castanopsis eyrei* (CE), *Castanopsis fargesii* (CF), *Keteleeria cyclolepis* (KC); bold values indicate relative frequency, relative abundance, and importance value of ECM fungi at the genus level; unbold values indicate relative frequency, relative abundance, and importance value of ECM fungi at the species level; *"–"* stands for absence.

Supplementary Table S6. Relative abundance of the dominant bacterial phyla (>1%).

Samples	Proteobacteria (%)	Acidobacteria(%)	Actinobacteria (%)	WPS-2 (%)	Chloroflexi (%)
PM	16.16±2.0b	21.47±2.06ab	9.06±9.06a	3.75±1.92ab	1.21±0.41b
CC	19.82±1.74ab	20.09±3.42ab	8.68±8.68a	1.38±0.43bc	1.86±0.67ab
CE	19.4±4.49ab	19.93±2.0ab	8.68±8.68a	5.29±1.51a	2.88±0.74a
CF	23.17±1.71a	23.75±1.2a	8.6±8.60a	0.87±0.46c	2.37±0.38a
KC	21.72±2.46ab	18.26±2.32b	9.75±9.75a	1.47±0.35bc	1±0.21b

Values are means ± standard deviation (n = 4). *Pinus massoniana* (PM), *Castanopsis carlesii* (CC), *Castanopsis eyrei* (CE), *Castanopsis fargesii* (CF), *Keteleeria cyclolepis* (KC); different letters indicate significant differences ($P < 0.05$) among forest types based on a one-way ANOVA followed by Tukey test.

Supplementary Table S7. Relative abundance of the bacterial genus (>1%).

Samples	***Acidothermus* (%)**	***Bradyrhizobium* (%)**	***Acidibacter* (%)**	***Candidatus_ Solibacter* (%)**	***Candidatus_ Koribacter* (%)**	***Roseiarcus* (%)**	***Bryobacter* (%)**
PM	6.94±0.29a	2.82±0.2b	3.65±0.57a	3.24±0.28b	1.57±0.35a	2.51±0.31a	2.6±0.18a
CC	5.31±0.5b	5.58±1.11a	3.34±0.63a	3.96±0.18ab	4.32±1.42a	1.65±0.24a	1.81±0.09a
CE	6.03±0.15ab	3.2±0.53ab	4.49±1.13a	3.51±0.14ab	2.64±0.47a	2.67±0.24a	2.07±0.25a
CF	3.53±0.17c	5.35±0.27ab	3.87±1a	4.5±0.25a	4.72±0.5a	2.14±0.22a	2.05±0.1a
KC	7.43±0.37a	5.11±0.58ab	4.97±0.77a	3.81±0.14ab	3.91±0.75a	2.12±0.3a	2.33±0.27a

Values are means ± standard deviation (n = 4). *Pinus massoniana* (PM), *Castanopsis carlesii* (CC), *Castanopsis eyrei* (CE), *Castanopsis fargesii* (CF), *Keteleeria cyclolepis* (KC); different letters indicate significant differences ($P < 0.05$) among forest types based on a one-way ANOVA followed by Tukey test.

Supplementary Table S8. Relationship of soil properties with RDA1 and RDA2 from the RDA analysis.

		pH	SOM	TP	TK	DOC	DON
ECM fungi	RDA1	0.4696	0.5477	0.0836	0.5812	0.3162	-0.6553
	RDA2	-0.8829	0.8367	0.9965	-0.8138	0.9487	0.7553
	R^2	0.7271	0.2445	0.6601	0.295	0.0793	0.4552
	p	**0.0005**	0.088	**0.001**	**0.0495**	0.5072	**0.007**
bacteria							
	RDA1	0.6017	-0.8684	-0.5809	0.9549	-0.8223	-0.7883
	RDA2	0.7987	-0.4959	-0.814	0.2969	-0.5691	-0.6153
	R^2	0.7432	0.3084	0.6292	0.9368	0.4582	0.2619
	p	**0.0005**	**0.048**	**0.0005**	**0.0005**	**0.002**	0.0605

Soil potential hydrogen (pH), organic matter (SOM), total phosphorus (TP), total potassium (TK), dissolved organic carbon (DOC), dissolved origin nitrogen (DON); Numbers in bold indicate significance at the $p < 0.05$ level.

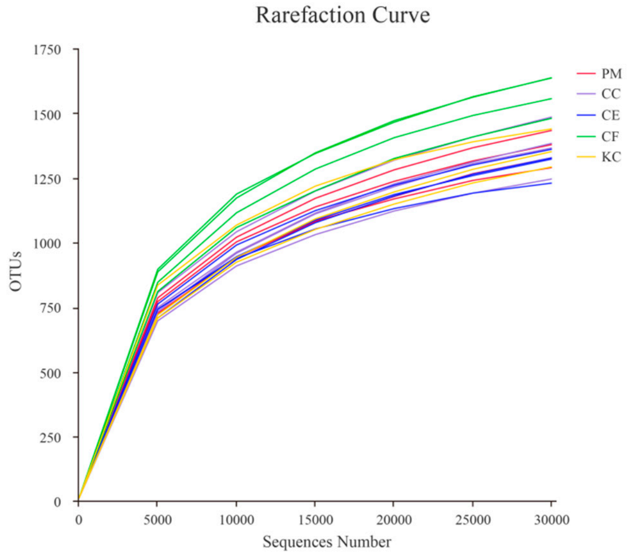

2. Supplementary Figures

Supplementary Figure S1. Bacterial community dilution curve of soil sample. The Alpha diversity index was used as the ordinate to draw the curve, and whether the volume of sequencing data was sufficient was assessed according to whether the curve was flat. *Pinus massoniana* (PM), *Castanopsis carlesii* (CC), *Castanopsis eyrei* (CE), *Castanopsis fargesii* (CF), *Keteleeria cyclolepis* (KC).

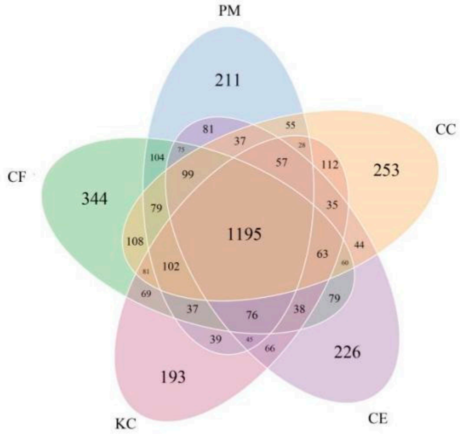

Supplementary Figure S2. Venn diagrams of OTU richness for PM, CC, CE, CF, and KC. *Pinus massoniana* (PM), *Castanopsis carlesii* (CC), *Castanopsis eyrei* (CE), *Castanopsis fargesii* (CF), *Keteleeria cyclolepis* (KC).
